# Transabdominal preperitoneal videolaparoscopic hernioplasty without mesh fixation

**DOI:** 10.1590/0102-672020260000014e1943

**Published:** 2026-07-10

**Authors:** Vitor NEVES, Gabriela LYONS, Fernando Athayde MADUREIRA

**Affiliations:** 1 Universidade Federal do Estado do Rio de Janeiro, Departament of Surgery – Rio de Janeiro (RJ), Brazil.; 2 Centro Universitário Souza Marques – Rio de Janeiro (RJ), Brazil.

**Keywords:** Inguinal Hernia, Laparoscopy, Unified Health System, Healthcare Costs, Hérnia Inguinal, Laparoscopia, Sistema Único de Saúde, Custos de Cuidados de Saúde

## Abstract

**Background:**

Laparoscopic inguinal hernia repair using the transabdominal preperitoneal (TAPP) technique requires a stapler for mesh fixation, making the method expensive.

**Aims:**

To demonstrate the feasibility of videolaparoscopic inguinal hernioplasty (TAPP) without mesh fixation in patients who are not stage 3 according to the European Hernia Society classification.

**Methods:**

Forty patients underwent videolaparoscopic inguinal hernioplasty (TAPP) with a polypropylene mesh and without fixation from October 2017 to October 2018. After the procedure, outpatient follow-up appointments were scheduled until the 6^th^ postoperative month. In June 2020, the patients were contacted by phone and a follow-up questionnaire was administered.

**Results:**

A total of 52 hernias were operated on, as 25% of patients had bilateral hernias. No patient presented with recurrence or chronic pain. Late in the course of the procedure, 72.5% of patients were contacted, with a mean follow-up of 27.2 months. The incidence of surgical site infection was 12.5%.

**Conclusions:**

The inguinal hernia repair by hernioplasty (TAPP) without mesh fixation in grade I and II patients (European Hernia Society) is feasible.

## ARTICLE HIGHLIGHTS

- Laparoscopic inguinal hernia repair without mesh fixation is safe and effective in selected small defects.
- Avoiding fixation devices significantly reduces procedural costs without compromising outcomes.
- This approach may improve access to minimally invasive surgery in public healthcare systems.
- The technique enables laparoscopic inguinal hernia repair in low-resource settings using fewer surgical materials.

## Summary

**CENTRAL MESSAGE:**

Inguinal hernia repair is the most commonly performed surgical procedure worldwide, with over 20 million operations carried out annually. Laparoscopic procedures are associated with reduced postoperative pain and a shorter recovery time, including a faster return to work. The transabdominal preperitoneal (TAPP) approach is one of the most widely used techniques for laparoscopic inguinal hernia repair and traditionally involves mesh fixation with disposable tackers.

**PERSPECTIVES:**

Laparoscopic inguinal hernia repair using the TAPP technique without mesh fixation in patients with European Hernia Society grade I and II defects is feasible. The technique showed no recurrences or chronic pain, high quality of life improvement, and a low rate of surgical site infections, supporting its feasibility and safety in selected patients. These findings suggest that, in carefully selected cases, TAPP inguinal hernioplasty without mesh fixation may be considered a safe and cost-effective alternative, particularly in resource-limited settings.

## VISUAL ABSTRACT

**Figure:**
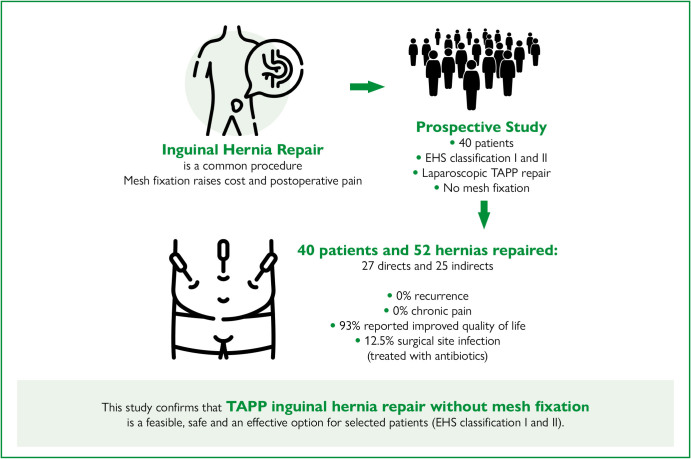

## RESUMO

**Racional:**

O reparo laparoscópico da hérnia inguinal pela técnica transabdominal pré-peritoneal (TAPP) demanda um grampeador para fixação da tela, tornando o método caro.

**Objetivos:**

Mostrar a viabilidade da hernioplastia inguinal videolaparoscópica (TAPP) sem fixação da tela nos pacientes que não são estagio 3 da classificação Sociedade Européia de Hérnias.

**Métodos:**

Foram operados 40 pacientes, entre outubro de 2017 até outubro de 2018 e submetidos a hernioplastia inguinal videolaparoscópica (TAPP) com tela de polipropileno e sem fixação. Após o procedimento, foram agendadas revisões ambulatoriais até o 6° mês pós-operatório. Em junho de 2020, foi realizado contato telefônico e aplicação de um questionário de acompanhamento dos pacientes.

**Resultados:**

Foram operados o total de 52 hérnias, pois 25% dos pacientes possuíam hérnias bilateralmente. Nenhum paciente apresentou recidiva ou dor crônica. Tardiamente, 72,5% dos pacientes foram contatados, com seguimento médio de 27,2 meses. A incidência de infecção do sítio cirúrgico foi de 12,5%.

**Conclusões:**

A correção da hérnia inguinal por hernioplastia (TAPP) sem a fixação da tela em pacientes graus I e II (Sociedade Européia de Hérnias) é factível.

## INTRODUCTION

Inguinal hernia repair is the most commonly performed surgical procedure worldwide, with over 20 million operations carried out annually^1^. Regardless of its origin or type, the definitive treatment for this type of hernia is surgical repair^15^.

Laparoscopic procedures are associated with reduced postoperative pain and a shorter recovery time, including a faster return to work^7,9,10,22^. Additionally, the laparoscopic approach provides improved identification of femoral and contralateral hernias that may not be detected during physical examination^2^.

The transabdominal preperitoneal (TAPP) approach is one of the most widely used techniques for laparoscopic inguinal hernia repair and traditionally involves mesh fixation with disposable tackers^17^. However, the use of such materials increases the overall cost of the procedure^21^. In healthcare settings with limited financial resources, such as hospitals within the Brazilian Public Health System (SUS), the high cost of surgical materials may limit access to minimally invasive techniques like laparoscopic hernioplasty.

The HerniaSurge Group, in its 2018 guideline, supports inguinal hernia repair without mesh fixation in several hernia subtypes, with the exception of large direct defects, classified as M3 by the European Hernia Society (EHS)^6,12^.

This study proposes a modified minimally invasive technique for inguinal hernia repair, aiming to maintain the benefits of laparoscopy, such as reduced postoperative pain, smaller incisions, and faster return to daily activities, while also offering an economically viable option for public healthcare institutions, particularly for one of the most prevalent surgical conditions in the general population^6,19^.

The aim of this study is to assess the feasibility of TAPP laparoscopic inguinal hernioplasty without mesh fixation in patients who are not stage 3 in the EHS classification. In addition, recurrence, quality of life, patient satisfaction, and the incidence of chronic pain and surgical site infection were evaluated.

## METHODS

From October 2017 to October 2018, 40 patients underwent laparoscopic inguinal hernia repair at Surgical Clinic A of the Gaffrée and Guinle University Hospital (HUGG).

The study included patients with primary (non-recurrent) inguinal hernias, either unilateral or bilateral, with a hernia ring smaller than 4 cm in diameter, who were clinically assessed during the initial outpatient evaluation. There were no age or sex restrictions. Patients with contraindications to general anesthesia or pneumoperitoneum were excluded.

All participants or their legal guardians provided written informed consent. The study was approved by the Research Ethics Committee of Gaffrée and Guinle University Hospital/HUGG/UNIRIO (Certificate of Presentation for Ethical Appreciation — CAAE: 73103317.4.0000.5258; Approval No. 3.783.213).

All procedures were performed by the same surgeon. Patients underwent laparoscopic inguinal hernia repair using the TAPP technique. Dissection and anatomical repair followed established surgical standards^3^. The mesh was placed without fixation, and no sutures, glue, or tackers were used.

### Transabdominal Preperitoneal (TAPP) operative technique

A 10 mm trocar was inserted through the umbilical incision for the laparoscope, along with two additional 5 mm trocars positioned at the same level as the optical port, 2 cm lateral to the rectus abdominis muscle, for the surgeon’s instruments. The procedure began with a peritoneal incision and dissection of the preperitoneal space.

The hernia sac was then dissected and its contents reduced. After confirming hemostasis, the hernia defect was classified according to the European Hernia Society (EHS) classification. A 15×10.5 cm polypropylene mesh was placed in the preperitoneal space.

The mesh was positioned to cover the anatomical sites prone to direct hernias (Hesselbach’s triangle), indirect hernias (internal inguinal ring), and femoral hernias (Gimbernat’s ligament), ensuring a minimum overlap of 4 cm beyond the margins of the hernia defect. The inferomedial edge of the mesh was placed 2 cm below the pubic symphysis, and the inferolateral edge rested on the psoas muscle. The superior border of the mesh was adjusted within the preperitoneal space to avoid folding or displacement.

Peritoneal closure was performed with a continuous suture using 3–0 polyglactin. All trocars were removed under direct visualization. The umbilical aponeurosis and skin were closed with monofilament sutures.

### European Hernia Society classification

In 2004, the EHS proposed a standardized classification system for inguinal hernias to be applied preoperatively or intraoperatively. This system aims to provide a more objective and straightforward method for hernia assessment, facilitating comparison of surgical outcomes^12^.

**Figure 1:**
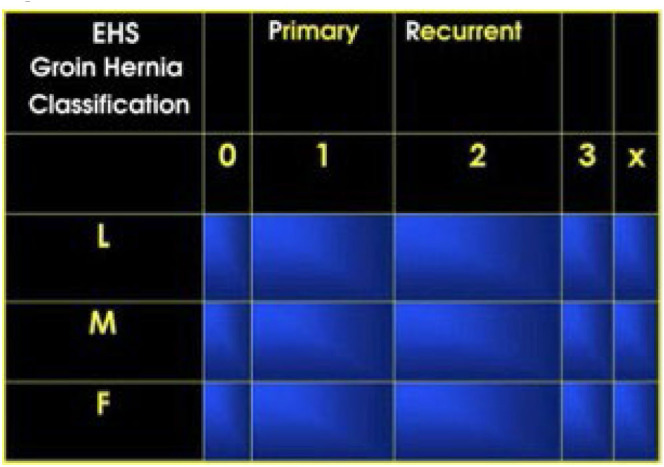
European Hernia Society (EHS) Classification for Inguinal Hernia^12^.

The size of the hernial orifice is classified as 1 (less than or equal to one finger, approximately 1.5–2.0 cm), 2 (one to two fingers), or 3 (equal or greater than three fingers), with the letter “x” used in cases where the size is unclear or not visualized. Anatomical location is indicated by the letters L (lateral or indirect), M (medial or direct), and F (femoral). Additionally, the letters P (primary) or R (recurrent) may be appended to further characterize the hernia^10^ (Figure 1).

### Postoperative follow-up

Postoperative evaluations were scheduled between the 5^th^ and 7^th^ days after surgery for suture removal, as well as on the 30th postoperative day and at 6 months following the procedure. Patients were allowed to resume daily activities as tolerated, with pain being the limiting factor; those without pain during activities were fully released. Return to work was generally permitted after two weeks, and resumption of intense physical activities was allowed between four and six weeks post-surgery.

In June 2020, all patients were contacted by telephone and asked the following questions:

1. On a scale from 0 to 10, how satisfied are you with the procedure?
2. Are you able to carry out your daily activities normally?
3. Regarding your quality of life, has the surgery resulted in improvement, deterioration, or no change?
4. How long after surgery did you feel able to perform physical activities such as running, cycling, or gym training?

This follow-up aimed to assess the presence of postoperative pain, the incidence of surgical complications, and patient satisfaction with the procedure.

### Statistical analysis

An initial exploratory analysis was conducted to identify inconsistencies in the database. Subsequently, a descriptive analysis was performed. Continuous variables with a normal distribution are presented as means and standard deviations, while those without normal distribution are described using medians, minimum, and maximum values. Normality was assessed using the Shapiro-Wilk test, with a significance level set at 5%. Categorical variables are expressed as absolute and relative frequencies. Statistical analyses were conducted using Statistical Package for the Social Sciences (SPSS) version 22, R version 4.01.1, and Microsoft Excel 2010.

## RESULTS

All patients were discharged on the morning following surgery. Of the 40 patients who underwent the procedure, a total of 50 meshes were placed using the TAPP technique without fixation (Table 1). Ten patients (25%) presented with bilateral inguinal hernias and underwent simultaneous bilateral repair during the same surgical session. Additionally, two patients (5%) had concomitant indirect and direct hernias on the same side of the inguinal region, resulting in a total of 52 hernias repaired (Table 2).

**Table 1:** Characteristics of the 40 patients undergoing transabdominal preperitoneal hernioplasty without mesh fixation.

	n (%)
Sex	
Female	4 (10.0)
Male	36 (90.0)
Age (years)	52.5±17.3
=19	1 (2.5)
20 to 59	22 (55.0)
=60	17 (42.5)
Smoking	
Yes	1 (2.5)
No	39 (97.5)
Follow-up (months)	5.0 [1.0–7.0]
Surgical site infection	
Yes	5 (12.5)
No	35 (87.5)

**Table 2:** Characteristics of the 52 corrected hernial defects, in the 40 patients who underwent transabdominal preperitoneal hernioplasty without mesh fixation.

	n (%)
Type of Hernia	
Direct	27 (51.9)
Indirect	25 (48.1)
EHS Classification	
M1	12 (23.1)
M2	17 (32.7)
L1	17 (32.7)
L2	6 (11.5)

EHS: European Hernia Society.

During outpatient follow-up, which extended to six months postoperatively, no cases of chronic pain or hernia recurrence were identified on physical examination.

Surgical wound infections were observed in five patients (12.5%) and were managed with oral antibiotic therapy (cephalexin); all infections occurred at the umbilical incision site. One patient developed a postoperative testicular hematoma, which resolved spontaneously. No other procedure-related complications were reported during the postoperative period.

After discharge, 29 patients (72.5%) were successfully contacted by telephone for long-term follow-up, while 11 patients could not be reached due to unavailable contact information provided at hospital admission. Follow-up contact occurred at a mean of 27.2 months (median: 27 months) after surgery. Patients were asked about persistent symptoms in the inguinal region; no cases of local bulging or chronic pain were reported.

**Figure 2:**
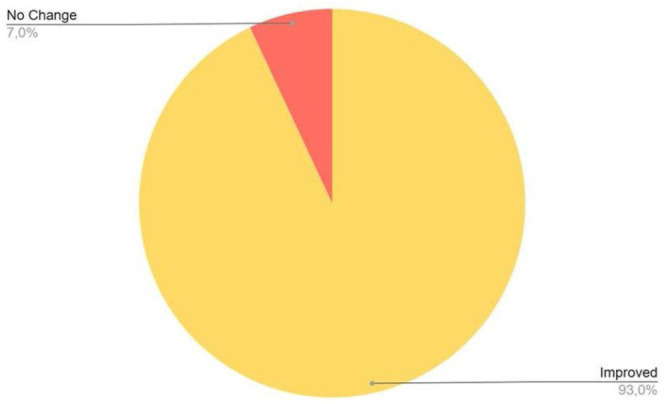
Quality of life reported by patients after undergoing transabdominal preperitoneal hernioplasty without mesh fixation (n=29).

**Figure 3:**
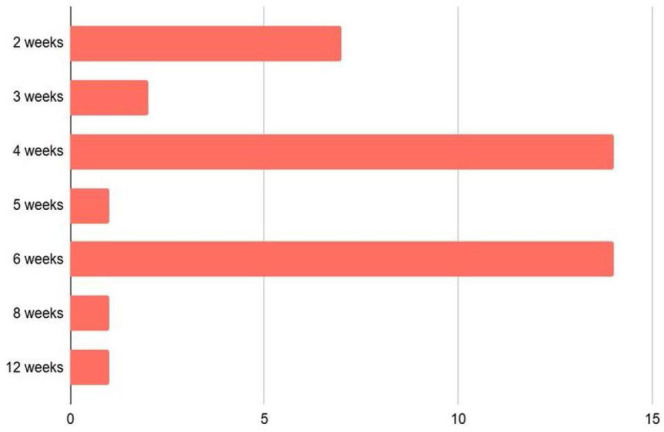
Time to return to all physical activities reported by patients after undergoing transabdominal preperitoneal hernioplasty without mesh fixation (n=29).

Regarding quality of life, 27 patients (93%) reported an improvement following the procedure, while two patients (7%) noted no difference, as they had experienced only mild symptoms preoperatively (Figure 2). All patients confirmed that they had returned to their routine daily activities (Figure 3).

## DISCUSSION

The most commonly employed laparoscopic techniques for inguinal hernia repair are the TAPP approach and the totally extraperitoneal (TEP) approach. Both methods have demonstrated high efficacy in addressing the pathology. Since Shulman’s report, the incidence of recurrence in tension-free repairs has been shown to be less than 1%^18^.

One of the main concerns in long-term patient follow-up is persistent inguinal pain. Chronic postoperative pain is a frequent complication of hernioplasty and can significantly affect patients’ quality of life, limiting their ability to work, sexual function, and social activities^4,14^. Fränneby et al. suggested that approximately one-third of patients experience local pain two to three years after undergoing a tension-free open hernioplasty, with around 6% reporting significant interference with daily activities due to pain^5^. According to a systematic review by Nienhuijs et al., chronic postoperative pain affects approximately 11% of patients undergoing inguinal hernia repair, regardless of the technique employed^13^.

It is well established that one of the main causes of chronic postoperative pain is the method of mesh fixation, whether by sutures, staples, or glue. During fixation, mechanical trauma or nerve fiber injury can occur, resulting in long-term pain.

In 2020, Torre et al. compared postoperative pain in patients undergoing TAPP hernioplasty, with one group receiving staple fixation and the other using a self-fixating mesh. In this study involving 52 patients, no statistically significant difference in postoperative pain was observed between the two groups^20^.

In 2012, Sajid et al. conducted a meta-analysis on laparoscopic hernioplasty with and without mesh fixation and concluded that omitting mesh fixation does not increase the risk of recurrence^16^. This finding has been supported by six additional randomized studies, which demonstrated that non-fixation of the mesh is not associated with higher recurrence rates.

In 2009, Kapiris et al. published a retrospective study analyzing 104 cases of inguinal hernioplasty performed using the TAPP technique without mesh fixation, and concluded that the method could be safely carried out without an increased risk of recurrence^8^. In 2017, Li et al. conducted a prospective, randomized study involving 100 patients and found that TAPP inguinal hernioplasty without mesh fixation is safe and effective for defects up to 4 cm^11^.

On average, 27.2 months after surgery, patients reported a prompt return to daily activities, improved quality of life, and a high level of satisfaction with the procedure. The results of the present study are consistent with findings reported in the literature. Although the initial sample size was limited, no cases of hernia recurrence were observed, supporting the feasibility and safety of performing TAPP inguinal hernioplasty without mesh fixation. Furthermore, no cases of chronic pain were reported beyond the sixth postoperative month, allowing patients to resume daily activities with improved comfort. Additionally, this technique was associated with high levels of patient satisfaction and perceived improvement in quality of life.

## CONCLUSIONS

Laparoscopic inguinal hernia repair using the TAPP technique without mesh fixation in patients with EHS grade I and II defects is feasible. These findings suggest that, in carefully selected cases, TAPP inguinal hernioplasty without mesh fixation may be considered a safe and cost-effective alternative, particularly in resource-limited settings.

## Data Availability

The datasets generated and/or analyzed during the current study are available from the corresponding author upon reasonable request.

## References

[1] Bay-Nielsen M, Kehlet H, Strand L, Malmstrøm J, Andersen FH, Wara P (2001). Quality assessment of 26,304 herniorrhaphies in Denmark: a prospective nationwide study. Lancet.

[2] Bisgaard T, Bay-Nielsen M, Kehlet H (2008). Re-recurrence after operation for recurrent inguinal hernia. A nationwide 8-year follow-up study on the role of type of repair. Ann Surg.

[3] Claus CMP, Oliveira FMM, Furtado ML, Azevedo MA, Roll S, Soares G (2019). Guidelines of the Brazilian Hernia Society (BHS) for the management of inguinocrural hernias in adults. Rev Col Bras Cir.

[4] Douek M, Smith G, Oshowo A, Stoker DL, Wellwood JM (2003). Prospective randomised controlled trial of laparoscopic versus open inguinal hernia mesh repair: five year follow up. BMJ.

[5] Fränneby U, Sandblom G, Nordin P, Nyrén O, Gunnarsson U (2006). Risk factors for long-term pain after hernia surgery. Ann Surg.

[6] HerniaSurge Group (2018). International guidelines for groin hernia management. Hernia.

[7] Hernandez-Rosa J, Lo CC, Choi JJ, Colon MJ, Boudourakis L, Telem DA (2011). Laparoscopic versus open inguinal hernia repair in octogenarians. Hernia.

[8] Kapiris S, Mavromatis T, Andrikopoulos S, Georgiades C, Floros D, Diamantopoulos G (2009). Laparoscopic transabdominal preperitoneal hernia repair (TAPP): stapling the mesh is not mandatory. JLaparoendoscAdvSurg TechA.

[9] Karthikesalingam A, Markar SR, Holt PJE, Praseedom RK (2010). Meta-analysis of randomized controlled trials comparing laparoscopic with open mesh repair of recurrent inguinal hernia. Br J Surg.

[10] Koning GG, Wetterslev J, van Laarhoven CJHM, Keus F (2013). The totally extraperitoneal method versus Lichtenstein’s technique for inguinal hernia repair: a systematic review with meta-analyses and trial sequential analyses of randomized clinical trials. PLoS One.

[11] Li W, Sun D, Sun Y, Cen Y, Li S, Xu Q (2017). The effect of transabdominal preperitoneal (TAPP) inguinal hernioplasty on chronic pain and quality of life of patients: mesh fixation versus non-fixation. Surg Endosc.

[12] Miserez M, Alexandre JH, Campanelli G, Corcione F, Cuccurullo D, Pascual MH (2007). The European hernia society groin hernia classification: simple and easy to remember. Hernia.

[13] Nienhuijs S, Staal E, Strobbe L, Rosman C, Groenewoud H, Bleichrodt R (2007). Chronic pain after mesh repair of inguinal hernia: a systematic review. Am J Surg.

[14] Palmqvist E, Larsson K, Anell A, Hjalmarsson C (2013). Prospective study of pain, quality of life and the economic impact of open inguinal hernia repair. Br J Surg.

[15] Rosenberg J, Bisgaard T, Kehlet H, Wara P, Asmussen T, Juul P (2011). Danish Hernia Database recommendations for the management of inguinal and femoral hernia in adults. Dan Med Bull.

[16] Sajid MS, Ladwa N, Kalra L, Hutson K, Sains P, Baig MK (2012). A meta-analysis examining the use of tacker fixation versus no-fixation of mesh in laparoscopic inguinal hernia repair. Int J Surg.

[17] Sanderson R, DE-Marchi DD, Cesário JCB, Sanderson LGD, Zilberstein B (2024). Quality of life using EURAHS-QOL scores after surgical treatment of inguinal hernia: laparoscopic transabdominal preperitoneal (TAPP) and Lichtenstein techniques. Arq Bras Cir Dig.

[18] Shulman AG, Amid PK, Lichtenstein IL (1992). The safety of mesh repair for primary inguinal hernias: results of 3,019 operations from five diverse surgical sources. Am Surg.

[19] Takahashi LKR, Arnoni LRR, Cardial DT (2017). Epidemiologua da hernia inguinal na população brasileira. J Coloproctol.

[20] Torre F, Madureira FAV, Hernández MAG (2020). Comparison of postoperative pain in laparoscopic inguinal hernia repairs by the transabdominal preperitoneal technique with self-gripping mesh versus tacker fixation. Int J Abdom Wall Hernia Surg.

[21] Wang WJ, Chen JZ, Fang Q, Li JF, Jin PF, Li ZT (2013). Comparison of the effects of laparoscopic hernia repair and Lichtenstein tension-free hernia repair. J Laparoendosc Adv Surg Tech A.

[22] Yang J, Tong DN, Yao J, Chen W (2013). Laparoscopic or Lichtenstein repair for recurrent inguinal hernia: a meta-analysis of randomized controlled trials. ANZ J Surg.

